# Characterization of Peanut Germin-Like Proteins, *AhGLPs* in Plant Development and Defense

**DOI:** 10.1371/journal.pone.0061722

**Published:** 2013-04-23

**Authors:** Tong Wang, Xiaoping Chen, Fanghe Zhu, Haifen Li, Ling Li, Qingli Yang, Xiaoyuan Chi, Shanlin Yu, Xuanqiang Liang

**Affiliations:** 1 Crops Research Institute, Guangdong Academy of Agricultural Sciences, Guangzhou, People's Republic of China; 2 Shandong Peanut Research Institute, Qingdao, People's Republic of China; 3 College of Life Science, South China Normal University, Guangzhou, People's Republic of China; Cankiri Karatekin University, Turkey

## Abstract

**Background:**

Germin-like superfamily members are ubiquitously expressed in various plant species and play important roles in plant development and defense. Although several *GLPs* have been identified in peanut (*Arachis hypogaea* L.), their roles in development and defense remain unknown. In this research, we study the spatiotemporal expression of *AhGLPs* in peanut and their functions in plant defense.

**Results:**

We have identified three new *AhGLP* members (*AhGLP3b*, *AhGLP5b* and *AhGLP7b*) that have distinct but very closely related DNA sequences. The spatial and temporal expression profiles revealed that each peanut *GLP* gene has its distinct expression pattern in various tissues and developmental stages. This suggests that these genes all have their distinct roles in peanut development. Subcellular location analysis demonstrated that AhGLP2 and 5 undergo a protein transport process after synthesis. The expression of all *AhGLPs* increased in responding to *Aspergillus flavus* infection, suggesting *AhGLPs'* ubiquitous roles in defense to *A. flavus.* Each *AhGLP* gene had its unique response to various abiotic stresses (including salt, H_2_O_2_ stress and wound), biotic stresses (including leaf spot, mosaic and rust) and plant hormone stimulations (including SA and ABA treatments). These results indicate that *AhGLPs* have their distinct roles in plant defense. Moreover, *in vivo* study of *AhGLP* transgenic *Arabidopsis* showed that both *AhGLP2* and *3* had salt tolerance, which made transgenic *Arabidopsis* grow well under 100 mM NaCl stress.

**Conclusions:**

For the first time, our study analyzes the *AhGLP* gene expression profiles in peanut and reveals their roles under various stresses. These results provide an insight into the developmental and defensive roles of *GLP* gene family in peanut.

## Introduction

Peanut (*Arachis hypogaea* L.) is one of the major worldwide oil crops. Peanut has very high nutritional and commercial value. However, the increase in its production is hampered by pathogens such as fungi, bacteria, viruses, insect pests and physiological stresses caused by chemicals and salt. It is estimated that yield losses of peanut are up to about 30% due to various disease and adverse physiological conditions [1]. So it is an urge task to identify and characterized resistant genes in peanut development and defense. An insight into functions and usage of resistant genes will make a great progress in peanut cultivation.

Germins and germin-like proteins (GLPs) are plant exclusive cupin subfamily water-soluble glycoproteins. Germin was first identified during wheat germination [2] and later was found to be oxalate oxidases (OXOs) [3]. Germins and germin-like protein subfamily are characterized by the presence of germin boxes (PHIHPRATEI) and a conserved cupin superfamily derived-motif [4], [5]. This motif is a conserved beta-barrel protein with a metal ion binding ability [6]. According to their sequence similarities and other characters, Germins and the *GLP* gene family are divided into two distinct group proteins. The first group named “the true germins” is only identified in “true cereals”, which contain barley, corn, oat, rice, rye and wheat. Members in this group have relatively homogeneous protein sequences [7] and always carry OXO enzyme activity. The second group is designated as germin-like proteins (GLPs), whose members show relatively high sequence divergence. Their amino acid sequence similarity to wheat germin varies from 30% to 70%. The second group contains more numerous members than the first group and only few of the second group members possess OXO activity.

*GLPs* are a large gene family and have a wide range of distribution among plants. Expressed sequence tags (ESTs) or genomic sequencing have identified more than 100 *GLPs*. In higher plant *Arabidopsis thaliana* genome, 27 *GLP* genes have been discovered [8], [9]. Also, 14 *GLP* genes in barley and 8 *GLP* genes in rice have been identified [10], [11]. In lower plants, Nakata et al. have identified 77 EST clones of GLPs from *Physcomitrella patens* [12]. *GLPs* also have a wide range of expression in various plant organs and developmental stages. *GLPs* have been identified to express in a variety of tissues such as roots, leaves and flowers [13]–[15]. The ubiquitous distribution of *GLPs* implies the *GLPs*' fundamental and indispensable functions in plants. And their expression in various organs suggests that *GLPs* may execute roles in the development of various plant organs.

GLPs play critical roles not only in plant development but also in plant defense responses. Several evidences have suggested the functions of GLPs in plant defense [16]. One is the observation of increasing expression of certain *GLPs* in various plants under stresses like fungal, bacteria, and viruse infections [5], [17]–[20], parasite attacks, insect invasions [21], chemical toxicities, salt pressures [22], [23] and drought stresses [24]. The other evidence of GLPs' roles in plant defense is the enhanced resistance of transgenic *GLP* plants to various stresses. For example, transformation of a wheat *GLP* into soybean, sunflower and tobacco provided them the resistance to *Sclerotinia sclerotiorum*. Transient overexpression of *GLPs* in Barley resulted in enhanced plant resistance to the powdery mildew fungus [5]. It is proposed that the mechanism by which GLPs function plant defense responses is associated to their enzyme activity of OXO and superoxide dismutase (SOD), which can generate H_2_O_2_ to influence plant defense. Additional enzyme activities of GLPs that may function in plant defense include ADP glucose pyrophosphatase/phosphodiesterase (AGPPase) [25] and serine protease inhibitors [26].

Expressed sequence tags (ESTs) have identified 8 *Arachis hypogaea* L. germin-like proteins (*AhGLPs*) in peanut [27]. The previous studies in our lab have revealed that the expression level of *AhGLP1*, originally named oxalate oxidase (OXO), significantly increased in *Aspergillus*- resistant peanut seeds after both drought and *A. flavus* treatments [28]. Due to GLPs' key functions in plant development and defense, we determined the expression of *AhGLPs* during peanut development and characterized the functions of *AhGLPs* both in biotic and abiotic stresses. Our results in this study suggest that *AhGLPs* express in various organs during peanut development and play important roles in plant defense. *AhGLPs* are valuable resistant genes that can be used in improving peanut pathogen resistance, thus increasing the yield in peanut cultivation.

## Results

### Identification of new *AhGLP* homologue genes

Through homology matrix, we have identified three new homologous members in the *AhGLP* family. According to their sequence similarity, we termed them as *AhGLP3a*, *AhGLP5a* and *AhGLP7a*. Sequence alignments revealed that these three new members all share more than 97% nucleotide homologies with other corresponding members in the family (Figure S1). The genetic polymorphism between *AhGLP3a* and *AhGLP3b* includes two point mutations and one deletion (Table 1), which lead to 3 amino acid changes. Seven nucleotide variations between *AhGLP5a* and *AhGLP5b*, and ten nucleotide variations between *AhGLP7a* and *AhGLP7b* were identified, respectively. The genetic variations relative to *AhGLP5a* and *AhGLP7a* are all point mutations. Among the seven point mutations between *AhGLP5a* and *AhGLP5b*, three were silent mutations, the other four ones result in amino acid variations. Similarly, among the ten point mutations between *AhGLP7a* and *AhGLP7b*, three were silent mutations, the other seven ones produce amino acid variations.

**Table 1:** Difference of closely related multiple *AhGLPs*.

Genes^a^	Nom.	Position	Type	Base Mutation	Protein Mutation
AhGLP3a vs AhGLP3b	1	107–108	Codon	AGC→-	Ser^36^→-
	2	124	Point	G→A	Val^43^→Met^42^
	3	562	Point	T→G	Phe^189^→Val^188^
AhGLP5a vs AhGLP5b	1	31	Point	G→A	Val^11^→Ile^11^
	2	90	Point	T→C	Tyr^30^ (Silent)
	3	370	Point	A→G	Ile1^24^→Val1^24^
	4	381	Point	T→C	Asp^127^ (Silent)
	5	429	Point	C→T	Tyr^143^ (Silent)
	6	553	Point	C→T	Pro^185^→Ser^185^
	7	597	Point	N^b^→G	*^199^→Glu^199^
AhGLP7a vs AhGLP7b	1	29	Point	T→A	Ile^10^→Asn^10^
	2	73	Point	G→A	Ala^25^→Thr^25^
	3	150	Point	T→C	Cys^50^ (Silent)
	4	208	Point	G→A	Ala^70^→Thr^70^
	5	241	Point	G→A	Ala^81^→Thr^81^
	6	295	Point	A→G	Thr^99^→Ala^99^
	7	307	Point	C→T	Rrg^106^ (Silent)
	8	363	Point	A→G	Pro^121^ (Silent)
	9	643	Point	G→A	Glu^215^→Lys^215^
	10	651	Point	N^b^→T	*^217^→Ile^217^

a *AhGLP3a* (GU457419.1), *AhGLP5a* (GU457421.1) and *AhGLP7a* (GU457423.1) were the known genes as reported by Chen et al (2011) in NCBI genebank, three newfound genes were designated AhGLP3b, AhGLP5b and AhGLP7b distinguished from AhGLP3a, AhGLP5a and AhGLP7a respectively.

b un-know bases in the nucleotide sequence of AhGLP5a and AhGLP7a with indefinable amino acids (*).

### Mapping of the developmental expression pattern of *AhGLP* mRNA in peanut

**Figure 1:**
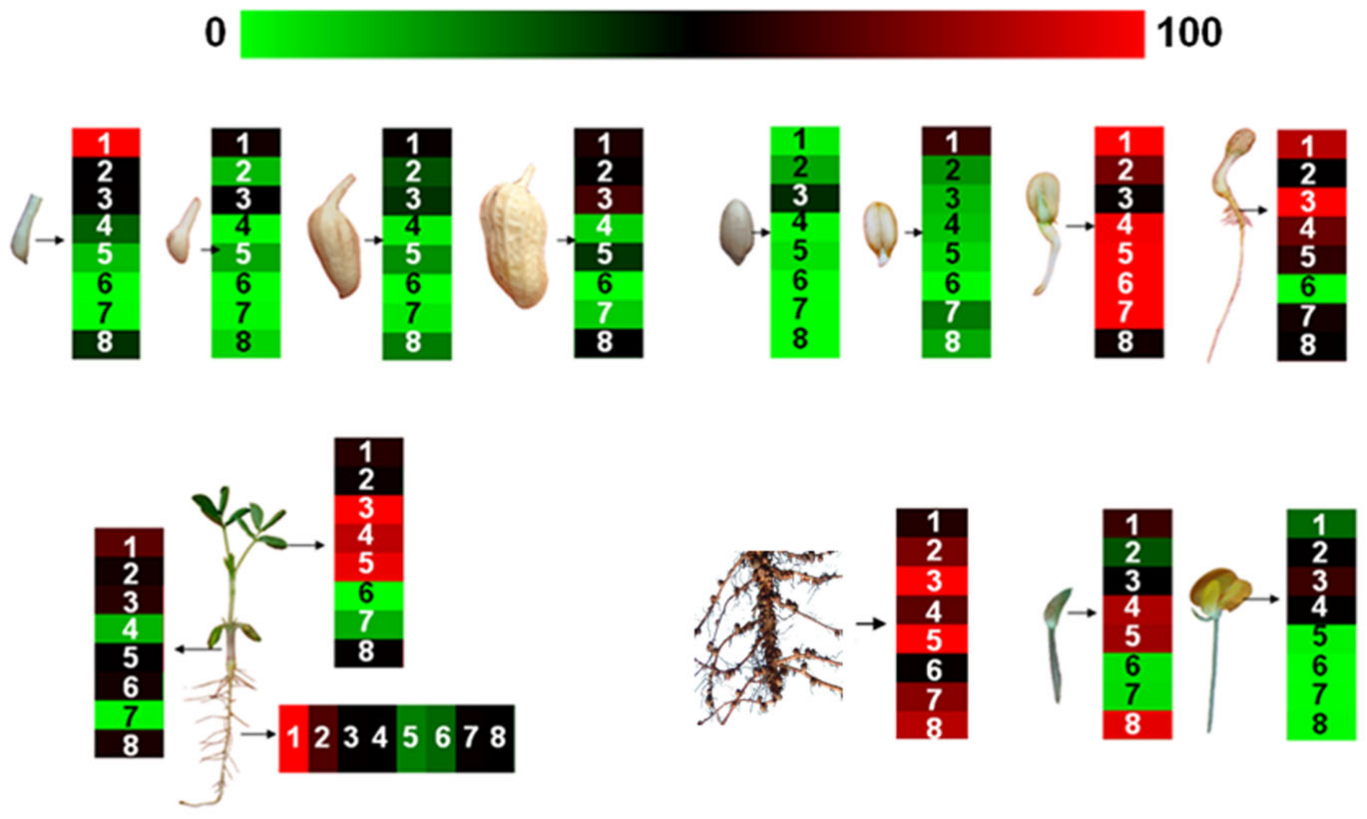
Expression patterns of peanut *AhGLP*s in various tissues/organs and developmental stages. Expression patterns of *AhGLP*s were examined in (A) developing pods at 1, 4, 15 and 35 days after penetration into soil, (B) seeds at 1 hour, 1, 3 and 5 days after germination, (C) roots, stems and leaves of 14-day-old seedlings, and (D) roots, flower buds and flowers during flowering. Transcript abundance detected using qRT-PCR was normalized to expression of *actin* gene. Numbers (1–8) inside boxes correspond to *AhGLP1*, *AhGLP2*, *AhGLP3b*, *AhGLP4*, *AhGLP5b*, *AhGLP6*, *AhGLP7b* and *AhGLP8*, respectively. Expression levels are color-coded on the bottom bar. Green color indicates the down-regulation of gene expression, red color indicates the up-regulation of gene expression, black indicates the RNA levels unchanged.

Expression pattern of the *AhGLP* family genes was first time examined in a variety of peanut tissues and various peanut developmental stages (Figure 1). During pods development (1–35d), *AhGLP1* mRNAs level was the highest 1 day after gynophore penetrated into under-soil, and decreased afterward. The mRNA of *AhGLP5* increased gradually during pods development. *AhGLP2*, *AhGLP3* and *AhGLP8* had similar expression pattern, which have the lowest level in the middle stages. Other three genes (*AhGLP4*, *AhGLP6* and *AhGLP7*) expressed at a very low level in these stages. During peanut seed germination, the expression level of all the *AhGLP* genes reached the hightest at day 3, except that of *AhGLP3* which was the hightest at day 5.

The mRNA level of the *AhGLP* family genes was determined in roots, stems, leaves, flower buds, and flowers. In the stems, *AhGLP1* had the highest mRNA level while *AhGLP4* and *AhGLP7* had the lowest expression level. In the leaves, *AhGLP3*, *AhGLP4* and *AhGLP5* were all highly expressed, while both *AhGLP6* and *AhGLP7* had a very low expression level. In the roots, *AhGLP1* also had the highest mRNA level. On the contrary, the mRNA level of *AhGLP5* and *AhGLP6* was very low. All the *AhGLP* family genes had a high mRNA level in old roots. The mRNA in flower buds decreased in the sequence of *AhGLP8, AhGLP4, AhGLP5, AhGLP1, AhGLP3, AhGLP2 and AhGLP6, AhGLP7*. In peanut flowers, only *AhGLP2*, *AhGLP3* and *AhGLP4* had a medium level expression. The various expression patterns of different *AhGLP* family genes suggest their various functions in different tissues and during various development stages.

### Determination of the subcellular localization of AhGLP proteins

**Figure 2:**
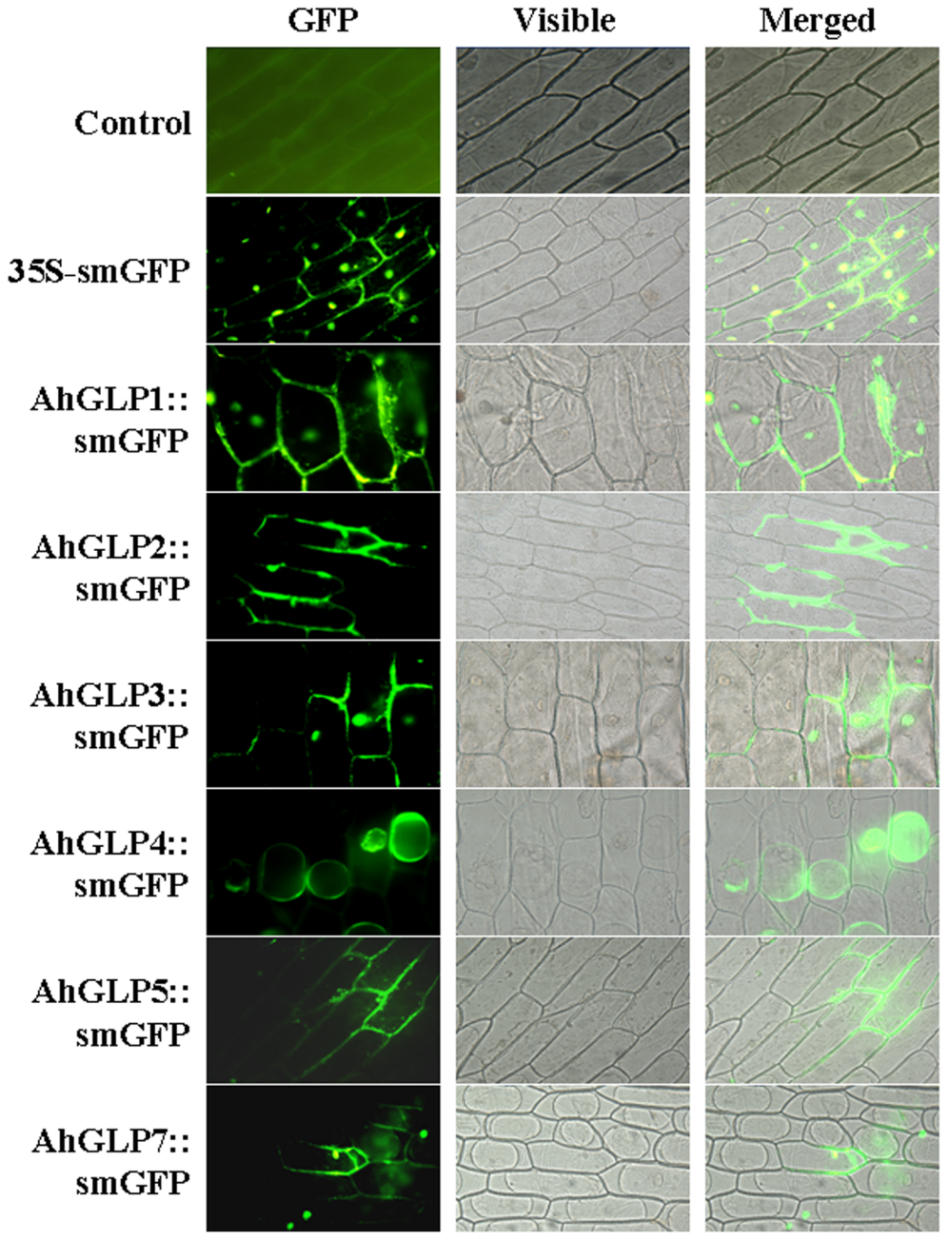
Subcellular localization of AhGLPs-GFP proteins in onion epidermal cells. Localization of AhGLPs-GFP fusion protein. Control: fluorescence of onion epidermal cells under empty vector. 35S-smGFP: onion epidermal cells expressing the *GFP* gene only driven by he 35S promoter. GFP fluorescence and differential interference contrast images and Visible/GFP merged images are shown from left to right.

The protein sequence bioinformation analysis showed that most AhGLPs contain putative extracellular localization signal peptides in their N-terminal. The prediction suggests that AhGLP proteins potentially target to the cell membrane or cell wall [27]. To confirm this possibility, the coding region of each *AhGLP* gene was respectively fused to the N-terminus of soluble modified *GFP* (smGFP) gene, whose expression was under the control of the constitutive *CaMV35S* promoter (*AhGLPs::smGFP*). Onion epidermal cell is a convenient system and widely used for analyzing protein subcellular location [29]. The obtained constructs, together with *non-AhGLP* control vector were then introduced into onion epidermal cells by *Agrobacterium*-mediated transient transformation. Subcellular localization of AhGLP proteins in onion (*Allium cepa*) epidermal cells, indicated by the GFP signal was observed under fluorescence microscopy. The results showed that compared to the control vector (35S-GFP), AhGLP1, 3 and 7 had similar subcellular location pattern being distributed both in cytoplasm and plasma membrane or cell wall whereas AhGLP4 was only localized in cytoplasm (Figure 2). On the contrary, AhGLP2 and 5 were only localized to the plasma membrane or the cell wall. The results suggest that most of AhGLPs can localize to the cell membrane or cell wall in consistence with AhGLPs having putative extracellular localization signal peptides in their N-terminal.

### Examination of the expression of *AhGLPs* in responding to *A. flavus* infection

**Figure 3:**
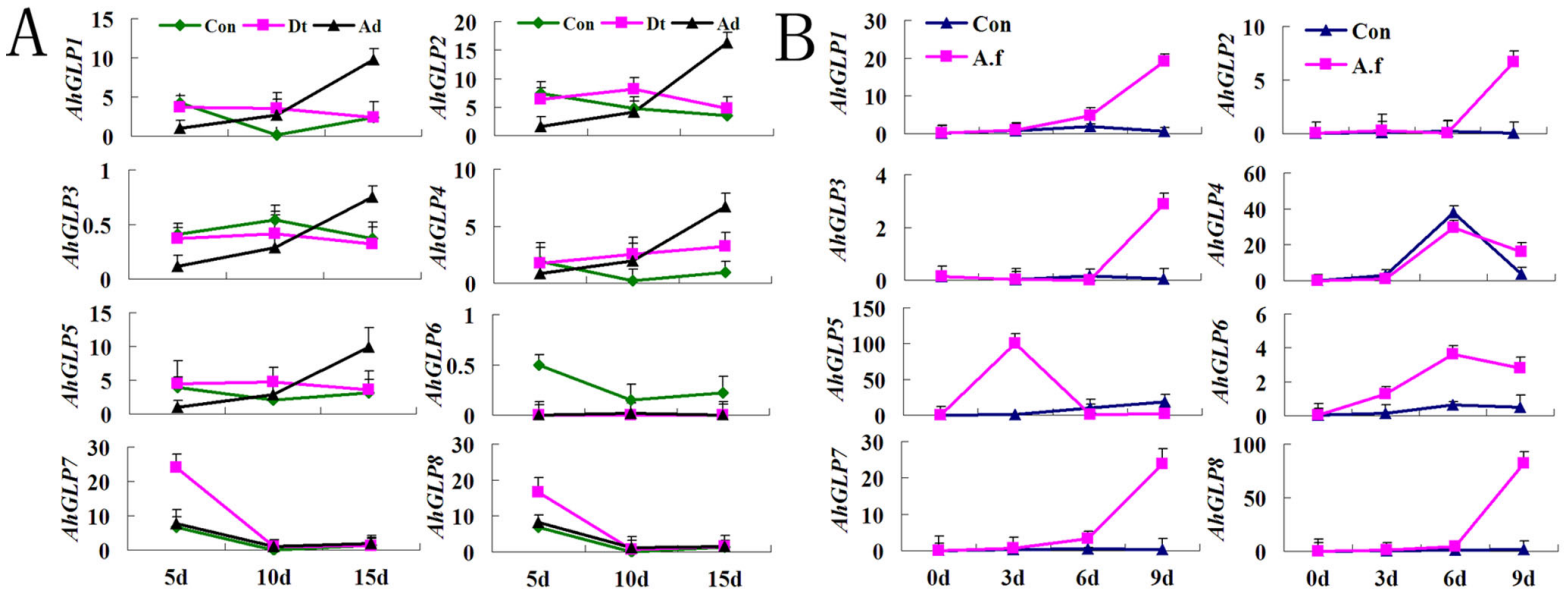
Expression of *AhGLPs* in response to *A. flavus* infection in pre - and post- harvested peanut seeds. A: Con: control; Dt: drought stress; Ad: *A. flavus* infection under drought stress condition; B: Changes of the expression of *AhGLP* family genes in damp-dry peanut seed with 20% RH (relative humidity) under *A. flavus* infection. Con: control; A. f: *A. flavus* infection.

*Aspergillus flavus* is a crop saprophyte that causes severe aflatoxin contamination to peanut seeds. To understand the possible roles of *AhGLPs* during *A. flavus* infection, we profiled the expression patterns of *AhGLP* genes in pre- and post-harvested peanut seeds after *A. flavus* infection. Compared to untreated control and drought-stresses treated pre-harvested seeds, according to T-test statistical analysis the expression level of *AhGLP1*, *2*, *3*, *4* and *5* were significantly up-regulated after *A. flavus* infections (Figure 3A). Conversely, the expression level of *AhGLP6, 7* and *8* did not change much after *A. flavus* infections. In addition, according to T-test statistical analysis drought treatments significantly decreased the expression of *AhGLP6*. This suggests that *AhGLP6* is the major gene responding to drought stresses.

For a further confirmation, the expression of AhGLPs in response to *A. flavus* infection was tested in post-harvested peanut seeds with 20% relative humidity (RH). Compared to untreated control, the expression level of all *AhGLPs* except *AhGLP5* was up-regulated 9 days after *A. flavus* infections (Figure 3B). And according to T-test statistical analysis, *AhGLP5* also significantly increased in day 3 after the infection. This suggests that *AhGLP5* is a quick responding gene in *A. flavus* infections. The data demonstrated that all *AhGLPs* are *A. flavus* repsonding genes and may have functions in peanut defense of *A. flavus* infections.

### Profiling of the expression of *AhGLPs* under various biotic, abiotic and hormone stresses

**Figure 4:**
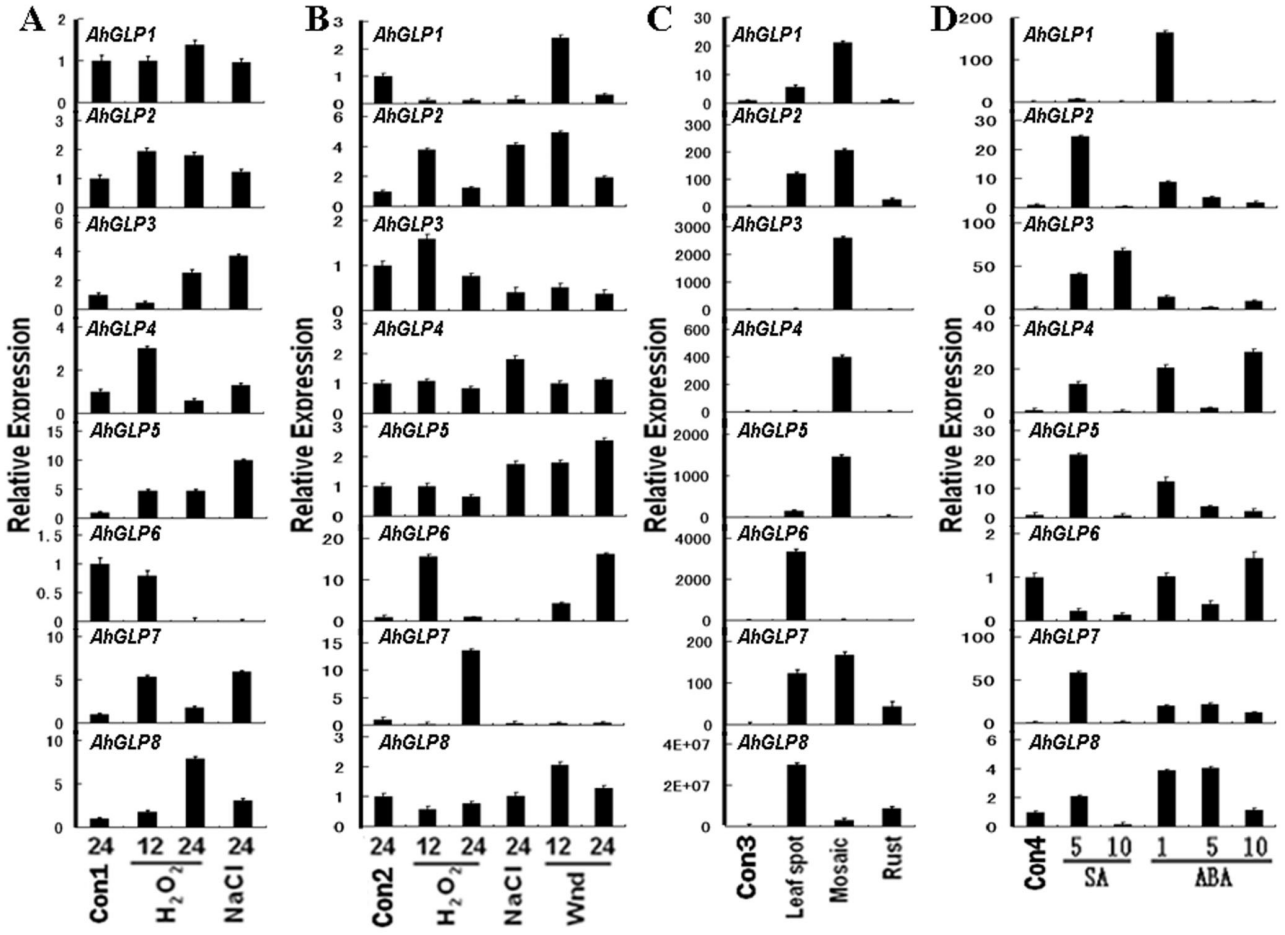
Differential expression of peanut *AhGLP* genes in response to various abiotic, biotic and hormone treatment conditions. *AhGLPs* transcript abundance was detected by qRT-PCR analysis. Con1 and Con2: control sample of seedling leaf and root to abiotic stresses, respectively; Con3: control of 100-day-old peanut plant leaf to biotic stresses; Con4: control of seedling leaf to hormone treatments; Wnd: wound treatment; Numbers below bars correspond to duration of treatments (h); SA: salicylic acid; ABA: abscisic acid.

In order to determine the roles of *AhGLPs* in plant defense, we examined the expression of *AhGLPs* responding to a variety of biotic, abiotic and hormone stress treatments. 7-day-old peanut seedlings were treated with various abiotic stresses including H_2_O_2_, salt and wound. Expression of *AhGLP*s in leaves and roots was then determined by quantitative RT-PCR analysis. Compared to mock-treated control, according to T-test statistical analysis H_2_O_2_ treatments significantly increased the expression of *AhGLP2*, *3*, *4*, *5*, *7* and *8* and decreased *AhGLP6* expression in roots after 12 and/or 24 hours (Figure 4A). According to T-test statistical analysis, NaCl treatments significantly increased the expression of *AhGLP3*, *5*, *7* and *8* and decrease *AhGLP6* expression in root in 24 hours (Figure 4A). In the leaves, the expression of *AhGLP*2, *6* and *7* was significantly up-regulated while *AhGLP1* expression significantly down-regulated 12 and/or 24 hours after H_2_O_2_ treatments according to T-test statistical analysis (Figure 4B). According to T-test statistical analysis, NaCl treatments significantly increased *AhGLP2* level in leaves. The expression of *AhGLP4* and *5* in leaves was up-regulated while the expression of *AhGLP1*, *3*, *6* and *7* in leaves was down-regulated after 24 hours under NaCl treatments (Figure 4B). The level of *AhGLP1*, *2*, *5*, *6* and *8* was up-regulated, while the level of *AhGLP3* and *7* was down-regulated 12 and/or 24 hours after wound treatments on the leaves (Figure 4B).

To demonstrate the functions of *AhGLPs* in plant disease defense, 80-day-old peanut plants were treated with a serial of biotic stresses including leaf spot, mosaic and rust. Then, the expression of *AhGLPs* mRNA in leaves was analyzed by qRT-PCR. According to T-test statistical analysis, leaf spot treatment significantly increased the expression of *AhGLP1*, *2*, *5*, *6*, *7* and *8* (Figure 4C). According to T-test statistical analysis, mosaic treatment significantly increased the expression of all *AhGLPs* excpet *AhGLP6* (Figure 4C). The level of *AhGLP2*, *7* and *8* was significantly up-regulated under rust treatments according to T-test statistical analysis (Figure 4C).

In order to examine the responses of *AhGLPs* to hormone stimulations, their mRNA level was determined in the leaves of 7-day-old peanut seedlings after the stimulations of hormones including salicylic acid (SA) and abscisic acid (ABA). According to T-test statistical analysis, the expression of *AhGLP2*, *3*, *4*, *5*, *7* and *8* was all significantly up-regulated 5 h after SA stimulation (Figure 4D). Among them, only *AhGLP3* expression kept going up, while the level of all others went down 10 hours after SA treatment. The level of *AhGLP6* kept going down after SA treatments. The expression of all *AhGLPs* except *AhGLP6* was up-regulated 1 h after ABA stimulation (Figure 4D). Except *AhGLP7* and *8*, the level of all other *AhGLPs* went down 5 hours after the treatments. The level of *AhGLP7* and *8* also went down in 10 h, while the expression of *AhGLP4* and *6* increased again 10 h after ABA treatments.

**Figure 5:**
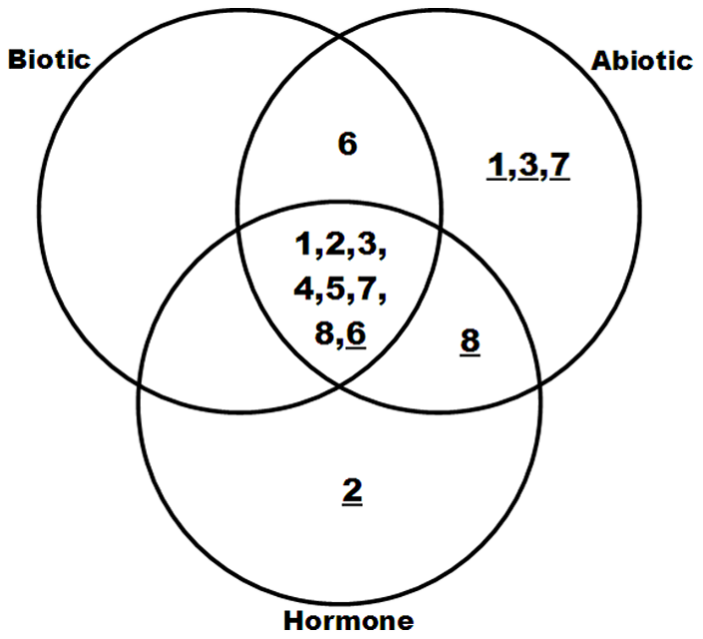
Venn diagram representing the expression profiles of *AhGLP* genes commonly or specifically regulated by various environmental stimuli, including plant hormones, abiotic stress and/or biotic stress in peanut leaves. 1, 2, 3, 4, 5, 6, 7, 8 were AhGLP1, AhGLP1, AhGLP2, AhGLP3, AhGLP4, AhGLP5, AhGLP6, AhGLP7 and AhGLP8, respectively. The underline numbers indicated down-regulated genes, and the numbers without underlines were up-regulated genes.

The results showed that various *AhGLP* gene expressions were induced in responding to different stresses stimulations. Their expressions induced were different and their expression had unique time windows under the stresses. This suggests that each *AhGLP* has their unique functions responding to various stresses in the plant defense. To have a comprehensive knowledge of how *AhGLPs* responding to various stimulations including hormone (including SA and ABA treatments), abiotic stress (including salt, H_2_O_2_ stress and wound) and biotic stress (including leaf spot, mosaic and rust), the overlap analysis of *AhGLP* genes response was performed (Figure 5). Most of the *AhGLP* genes including *AhGLP1*, *2*, *3*, *4*, *5*, *7* and *8* had increasing expression in responding to all of these three stimulations. Whereas, *AhGLP6* expression was down-regulated in responding to most of the stimulations except wound and leaf spot treatments. Additionaly, the expression of *AhGLP1*, *3* and *7* was down-regulated under abiotic stresses. It is also interesting to note that all peanut *GLP* family genes primarily had a positive-response to biotic stresses through an increasing mRNA expression.

### Verification of the salt tolerance functions of *AhGLPs* in transgenic *Arabidopsis thaliana*

Due to the difficulty of stable transformation of peanut, to study the functions of *AhGLPs in vivo* we engineered transgenic *Arabidopsis* plants constitutively expressing *AhGLPs* through the cauliflower mosaic virus 35S constitutive promoter. After selection with 25 mg/L hygromycin at the T2 generation and confirmation by RT-PCR with the *AhGLPs*-specific primers, at least three independent transgenic lines carrying a single copy of the each *AhGLP* insertion were found to constitutively express the *AhGLP* genes, which could not be detected in wild-type *Arabidopsis*.

**Figure 6:**
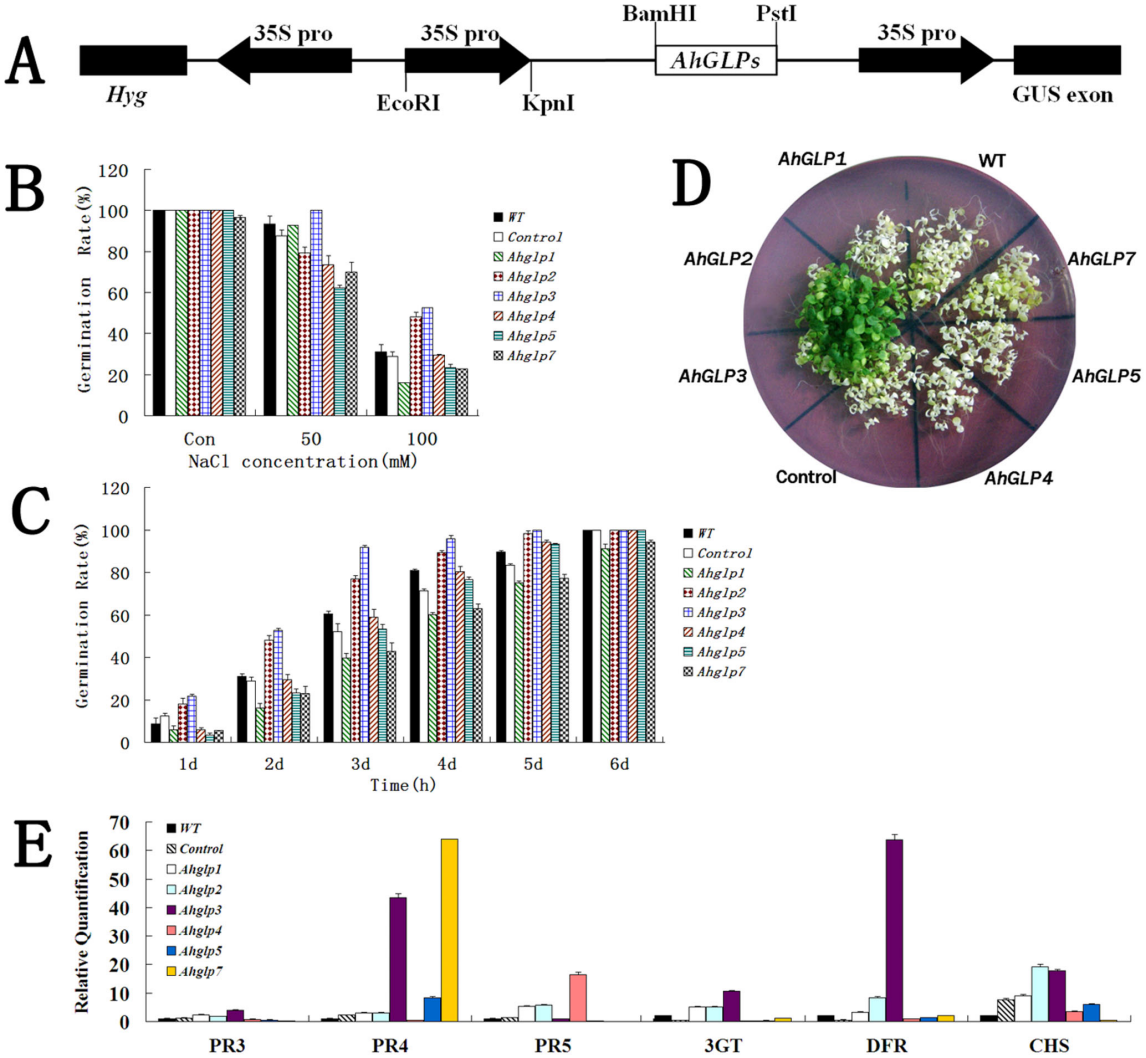
Overexpression of *AhGLP*s and salt tolerance analysis in transgenic *Arabidopsis thaliana*. WT: wild type; control: The modified pCAMBIA1301 inserted with 35S only was used as negative control. (A): Diagrams of the constructs (the pCAMBIA1301-35S- *AhGLP1*, *2*, *3*, *4*, *5* and *7*) used for *Agrobacterium tumefaciens*-mediated transformation of *Arabidopsis*. (B): Effects of different NaCl content (0, 50 and 100 mM) on the germination of transgenic *Arabidopsis* seeds for 5 days after germination; (C): Comparison of germination rates and percentages of seedlings with green cotyledons between transgenic lines and wild-type plants under 100 mM NaCl stress. (D): The seeding cultivated on 1/2 MS agar plate containing 50 mM NaCl for 15 days. (E): Transcript analysis of *AhGLPs*-activated defense related genes (DFR, CHS, 3GT, and AtPR3, 4 and 5) in transgenic *Arabidopsis* plants.

In previous study, NaCl treatments significantly increased the expression of several *AhGLPs*. To verify the functions of these *AhGLPs in vivo*, we challenge these transgenic *Arabidopsis* lines of T3 progeny with salt stresses. The result of the 3 independent lines were similar, here we choose to propose the data of one typical line. Wild type and transgenic *Arabidopsis* seeds of T3 progeny were grown under untreated and salt treated conditions, and their germination rate was calculated. Under untreated conditions, the germination rates of wild type, empty vector transgenic positive control and *AhGLP* transgenic *Arabidopsis* seeds were not significantly different (Figure 6B control). After treatment with various concentrations of NaCl, according to T-test statistical analysis both *AhGLP2* and *3* overexpression *Arabidopsis* seeds displayed significantly higher germination rates than other seeds including wild type and empty vector transgenic control seeds (Figure 6B). The highest germination rates of *Arabidopsis* seeds overexpressing *AhGLP2* and *3* maintained throughout the time points (from day 1 to day 6) (Figure 6C). To further confirm the salt tolerance roles of *AhGLP2* and 3, *Arabidopsis* seedlings were treated with 100 mM NaCl for 15 days. When all other *Arabidopsis* seedlings grew badly with yellow leaves, the seedlings of *AhGLP2-* and *3*- overexpressing *Arabidopsis* grew very well with green leaves (Figure 6D). These results suggest that *AhGLP2* and *3* have roles in salt tolerance.

To define a possible correlation between salt tolerance and defense gene expression, we analyzed the transcript levels of defense genes (*PR3* to *5*) and antioxidant-related genes (*CHS*, *DFR* and *3GT*) in transgenic *Arabidopsis* plants through qRT-PCR. After T-test analysis, the expression of all genes except *PR3* was significantly up-regulated in *AhGLP2* overexpression *Arabidopsis* plants. After T-test analysis, the expression of all genes except *PR5* was significantly up-regulated in *AhGLP3* overexpression *Arabidopsis* plants (Figure 6E). This result pointed out that the salt tolerance of *AhGLP2-* and *3* have a positive correlation to defense gene expression level. The expression of *PR4* was also up-regulated in *AhGLP5*- and *7-* transgenic *Arabidopsis*, indicating their correlation. PR5 level was obviously up-regulated in *AhGLP4* overexpression *Arabidopsis*, suggesting their positive correlation. Thus, the increasing level of defense genes in *AhGLP2* and 3 transgenic *Arabidopsis* may partly contribute to their resistance to salt stresses.

## Discussion

A great deal of GLP family members have been identified and divided into 5 subfamilies including bryophyte GLP subfamily, gymnosperm GLP subfamily, “ture gemin subfamily”, GLP subfamily 1, GLP subfamily 2 and GLP subfamily 3. Guo et al [27] reported the presence of eight GLP members in peanut based on the analysis of EST database and divided them into three classes including GLP subfamily I (AhGLP3a), GLP subfamily II (AhGLP2, 6 and 8) and GLP subfamily III (AhGLP1, 4, 5a and 7a) according their protein sequence feature. The sequence identities of these subfamily members ranged from 31.3% to 72.0%. In this study, we have identified three new *AhGLP* homologues named as *AhGLP3b*, *AhGLP5b* and *AhGLP7b*. They all showed highly homology compared with the corresponding *AhGLP* homologues like *AhGLP3a* (99.1%), *AhGLP5a* (98.6%) and *AhGLP7a* (97.7%) (Additional File 3). It has been found that the closely related GLPs including *AtGLP2a/b* (97.7% identity) and *AtGLP3a/b* (99.5% identity) in *Arabidopsis* had very similar functions [8]. So, we deduced that these closely related *AhGLPs* could also have the similar functions in peanut.

Although the spatiotemporal expression of germins and *GLPs* in different plant species has been characterized [30]–[33], their pattern in peanut remained unclear. In this study, we surveyed the transcript accumulation of the eight *AhGLP* genes across a wide range of tissues/organs and developmental stages of peanut through qRT-PCR analysis. Our results for the basal expression patterns of each of the peanut *GLP* genes (Figure1) suggest that each *AhGLP* has its distinct expression pattern in different tissues and stages, indicating that the spatiotemporal regulation of their expression is distinct. Furtheremore each *AhGLP* had its distinct function during peanut development. For example, *AhGLP8* and *AhGLP4* had a very high level in flower buds and significantly decreased in flowers, suggesting that these two genes may function in peanut flowering. The low expression level of *AhGLP6* and *AhGLP7* in both flower buds and flowers indicates that the two genes may not function in flowering. Also, the expression pattern of *AhGLP1* suggests its potential roles in roots and pods development and seed germination. Besides, *AhGLP3*, *AhGLP4* and *AhGLP5* may function in leaf development and *AhGLP3* may play a role during seed germination. Moreover, the specificity of *AhGLPs* expression paved the way that *AhGLPs* can be used as markers of various developmental stages in future.

It has been identified that in wheat about 40% germins are associated with cell wall and critical for development [34]. The N-terminal signal peptide in Germin and GLPs has been proved to help their secretion from the cell [35]. Cellular localization studies also confirmed the association of GLPs with the cell surface [20], [32]. In rice, a high abundance of GLPs has been shown to locate in epidermal cells [36]. Sequence analysis identified a signal peptide in AhGLPs N-terminal region, suggesting the possibility of AhGLP apoplastic or plasma membrane localization [27]. Our results showed that all AhGLPs are distributed at plasma membrane or cell wall (Figure 2). This confirmed their N-terminal signal peptide's functions. Among these AhGLPs, only AhGLP2 and 5 were excluded from the nucleus. The results suggest that AhGLP2 and 5 may perform a protein translocation process after synthesis.

The expression of GLPs and germin has been shown to be differentially regulated responding to pathogen attack in several plant species such as barley [5], rice [20], grapevine [31] and *Arabidopsis* [37]. They are expressed in a diverse range of tissues and have inkling of the broad spectrum of defensive activities in host-pahogen interaction [35]. It is worth mention that the overexpression of barley OXO gene results in enhanced resistance to the *Sclerotinia* minor in peanut [38]. In contrast, transient silencing of some barley GLP subfamilies increased the susceptibility to powdery mildew fungus [5]. Recently, our previous proteomics study revealed that the level of AhGLP1 protein, originally named as OXO, was positively correlated to *A. flavus* infection in pre-harvested resistant peanut seed [28]. In our study, the expression of most *AhGLP* family genes was also induced significantly when attacked by fungal pathogens (leaf spot, *A. flavus*) and bacteria (Rust disease) in peanut leaves (Figure. 4B). This suggests that AhGLPs play important roles in pathogen defense. To further understand the molecular function of *AhGLP* family genes in response to *A. flavus* infection, the expression patterns were analyzed in pre- and post-harvest peanut seeds during *A. flavus* invasion, respectively (Figure 3A and B). The results showed that all *AhGLP* mRNAs might response to *A. flavus* infection in seeds. Like GLPs in other plant species, our results suggested that AhGLPs are also broad spectrum and effective defense proteins against multiple pathogens.

Plants have a variety of strategies to adapt to unfavourable environmental conditions including various abiotic and biotic stresses [39]. Many studies have indicated that the germin and GLPs play important roles in resistance to various abiotic and/or biotic stresses [35]. In some model crops and plants, such as wheat, barley, rice and *Arabidopsis*, a number of *GLP* genes have been shown to function in response to stresses mainly thought their SOD [33], [40], [41], OXO [42] and AGPPase enzyme activity [25]. Although some of peanut *GLP* gene sequences were present in the GenBank database, so far only AhGLP2 has been identified to have SOD enzyme activity [27]. In this study, we analyze the differential expression of *AhGLPs* between peanut roots treated with different abiotic (NaCl, H_2_O_2_, wound) stresses and untreated control (Figure 4A). Same analysis was performed in the leaves. Six barley HvGER family genes [5] appeared to participate in multiple abiotic stress responses in leaf and root. Similarly, this analysis showed that all the peanut *GLP* genes were significantly differentially expressed under at least one of the abiotic stresses in leaves. Moreover, in roots, only *AhGLP2*, *3*, *4*, *5*, *7* and *8* changed their expression after NaCl and H_2_O_2_ treatments. These results indicated that the expression of peanut *GLP* genes has tissue-variability and AhGLPs are involved in different regulation pathways in response to abiotic stresses. Phytohormones are important factors that participate in plant gene regulation networks involving abiotic and biotic response and tolerance [43]. The transcript level of some *GLPs* are enhanced or suppressed after application of phytohormones including salicylic acid (SA) [21], [31], [44] and abscisic acid (ABA) [45]. In this study, all the peanut *GLP* genes significantly changed their expression level under treatment of exogenous SA and ABA (Figure 4C). Among them, *AhGLP6* expression was inhibited, the expression of all other seven members was induced. The result suggests that they might play roles in SA/ABA-dependent signaling transduction pathways during abiotic and biotic stress responses. Germin and GLPs are multi functional proteins, and many expression studies of these genes have shown crosstalk between various stimuli such as biotic, abiotic and hormone [5], [20], [31]. Our overlap analysis also found relation between tissue-/developmental stage-specific expression pattern and stress responses of peanut *GLP* genes (Figure 1, 2 and 3). It is noteworthy that *AhGLP* family genes showed broad spectrum stress responses mostly in peanut leaves. However, only *AhGLP6* showed response to specific stimuli (Figure 3). GLPs resistance may be broad spectrum and effective against various environmental stimuli [20], [37]. These commonly regulated *AhGLPs* will provide some promising candidate genes for genetic engineering for improving crop resistance to different stresses.

*GLP* plays important roles in salt resistance. It has been proved that in responding to salt stress *GLP* expression increased in barley roots [22]. Moreover, salt stress can prolong the expression of *GLP* in barley [46]. Also, the *GLP* genes were proved to response to salt stress in soybean [47]. GLP proteins were gradually up-regulated during the period of salt treatment in wheat leaves [48]. Proteome analysis demonstrated that germin-like protein increased significantly in response to salt stress in the tobacco leaves [49]. In respond to salt stress, not only the expression of *GLP* but also the expression site changes. In salt-stressed wheat embryos, germin mRNA change their location to coleoptile cells instead of its original site, coleorhiza tissue [50]. In a moss, it is proposed that dissociation of GLP protein from the cell wall into the medium in the cells caused the induction of *GLP* gene by salt stress during the logarithmic phase [51]. Recently, it has been discovered that several other plant *GLP* genes can enhance the tolerance to salt stresses in transgenic plants [52]. After salt treatments, the expression of *AhGLP3*, *5* and *7* significantly increased in root. However, in the leaves only *AhGLP2* expression increase obviously in response to salt treatment. The results suggest these 4 *AhGLPs* may function under salt stresses. To confirm this possibility, we engineered *AhGLP* transgenic *Arabidopsis* plants and challenge the plants with salt stresses. Both *AhGLP2-* and *3-* overexpression *Arabidopsis* seeds displayed significantly higher germination rates than other seeds (Figure 6B). Moreover, the highest germination rates maintained throughout the time points (from day1 to day6) (Figure 5C). After treated with 100 mM NaCl for 15 days, only the seedlings of *AhGLP2-* and *3-*overexpressing *Arabidopsis* still grew very well with green leaves (Figure 6D). These results suggested that both *AhGLP2* and *3* genes were involved in the salt stress response and tolerance in plants.

Salt stress could accelerate production of active oxygen species (AOS) and subsequently cause oxidative damage in plants. Antioxidants, therefore, are elements of the salt stress response in a manner similar to general stress responses in plants [53], and various types of SODs are thought to have important roles in controlling oxidative stress [53]. Thus, one of possible explanation for the tolerance function of AhGLP-2 and 3 in *Arabidopsis* could be the SOD enzymatic activity, although only AhGLP2 have been identified to have SOD activity [27]. Moreover, it has been reported that the high level of flavonoid may be related to high salt stress tolerance through scavenging of stress-induced AOS [54]. In this study, *Arabidopsis* plants overexpressing *AhGLP2* and *3* enhanced the transcript levels for flavonoid biosynthetic genes including *DFR*, *CHS* and *3GT* than control plants. Furthermore, the expression of PR proteins (AtPR3, 4 and 5) in transgenic *Arabidopsis* with *AhGLP* was enhanced diversely. This result is consistent with the studies by Knecht [37], in which *BvGLP1* overexpression in *Arabidopsis* induced SA- and JA-dependent *PR* genes transcripts. These transgenic lines could be better protected through activation of the defense genes even in the absence of the pathogens. This strongly suggests that *AhGLP2* and *3* may play importantly functional roles in plants by specifically regulating the expression of a set of plant defense-related genes. The function of *AhGLPs* under pathogen attack will be further studied.

## Conclusions

In general, expression patterns of 8 peanut *GLP* genes were analyzed in different tissues and stages. Their expression in response to various biotic stresses and abiotic stresses, and plant exogenous hormone treatments was also analyzed through qRT-PCR. The results revealed that expression levels of *AhGLP* family genes are varied greatly in different tissue*-*/developmental stage and various stresses. Moreover, six Ah*GLP*s have been isolated and were used for expression in *Arabidopsis* and subcellular localization analysis in onion cells. The results indicated that *AhGLP2* and *3* might be salt stress response and tolerance genes and most of the *GLP* genes located plasma membrane or cell wall.

## Materials and Methods

### Ethics Statement

No specific permits were required for the described field studies. No specific permissions were required for these locations and activities. The location is not privately-owned or protected in any way and the field studies did not involve endangered or protected species.

### Plant material and sampling

A cultivar peanut (*Arachis hypogeae*. L) YJ-1 with resistance to *Aspergillus flavus* infection was provided by Crops Research Institute, Guangdong Academy of Agricultural Sciences (GDAAS, China). The seeds were surface sterilized using 70% (v/v) ethanol for 2 min, and then sown in pots of compost soil in a greenhouse under white fluorescent light (16 hr light/8 hr dark) at 30°C and 70% relative humidity. After germination, seedlings and plants were randomly divided into several groups (each containing six samples) and subjected to different stress treatments as explained below. Mature leaves, roots, stems, various stage panicles and seeds were collected. Roots from 7-day-old seedlings were also harvested.

For salt and H_2_O_2_ stress treatments, 7-day-old light-grown peanut seedlings were watered with solutions of 100 mM NaCl and 100 mM H_2_O_2_, respectively. After 12 and 24 h, the leaves and roots were collected. Likewise, 7-day-old seedlings were incubated with 100 µM ABA-solution and 50 µM salicylic acid (SA)-solution, respectively. And the leaves were collected after 1, 5 and 10 h. For the wound treatment, primary leaves of 7-day-old seedlings were rubbed gently with fine sandpaper and samples were collected after 12 and 24 h. Untreated samples were collected as controls at the same time points.

For peanut leaf disease treatments, 35-day-old plants were sprayed or inoculated with a spore suspension of leaf spot, mosaic and rust [55]. Triplicate samples of control and infected peanut leaves were collected. The post- and pre-harvested peanut seeds of cultivar YJ-1 were challenged with *A. flavus* according to the method of Liang [56] and Wang [28], respectively. The post-harvest seeds were collected 0, 3, 6 and 9 days after *A. flavus* infection, and the pre-harvest seeds were sampled 5, 10, 15 and 20 days after treatments with both drought and *A. flavus* stresses. All plant materials were snap frozen in liquid nitrogen and stored at −80°C.

### RNA isolation and purification

Total RNA was isolated from peanut tissues and *Arabidopsis* leaves (wild-type and *AhGLPs*-transgenic) using TRIzol reagent (Invitrogen, Carlsbad, CA) according to manufacture's instructions. All samples were collected from three biological replicates [28]. All RNA extracts were treated with RNase-free DNase I (Takara, Dalian, China) then cleaned up with RNeasy Cleanup Kit (Qiagen, Beijing, China). RNA concentration and quality were assessed by Nano Drop ND-100 spectrophotometer (Nano Drop Technologies Inc., Delaware, USA) and electrophoresis on 1% agarose gel. The obtained RNA was stored at −80°C.

### Quantitative real time RT-PCR

All qRT-PCRs were performed as described previously [28]. 4 µg of total RNA was reverse transcribed to cDNA using PrimeScript II 1^st^ Strand cDNA Synthesis kit (TaKaRa, Dalian, China) according to the manufacturer's protocols. Quantitative real-time RT-PCR was performed with SYBR® Premix Ex Taq™ II kit (TaKaRa, Dalian, China) in LightCycler 480 instrument (Roche, Germany) equipped with Light-Cycler Software version 1.5 (Roche, Germany) according to the manufacturer's instructions [57].

All the primers specific for peanut *AhGLPs* and *Arabidopsis* stress-related genes were designed using the Primer version 5.0 (PREMIER Biosoft International) and listed in Table S1. The *18S rRNA* and *actin* gene were used as internal controls for calculating relative transcript abundance in peanut and *Arabidopsis*, respectively. All real-time PCR reactions were repeated three times. The relative quantification of RNA expression was calibrated using formula 2^−ΔΔCt^ method [58]. The mean of technical replicates was presented in the results. T-test analysis was performed to determine the statistical significance.

### Generation of *AhGLPs*-transgenic *Arabidopsis* plants

The full-length coding sequence (ATG to TAA) of *AhGLP1*, *2*, *3*, *4*, *5* and *7* were amplified by PCR with the gene-specific primers (Table S2). These PCR products were inserted into pCAMBIA 1301 vector, which expression was under the control of Cauliflower mosaic virus (CaMV) 35S promoter. After sequencing confirmation, the recombinant plasmids were introduced into *Agrobacterium tumefaciens* GV3101 through the freeze-thaw method and then introduced into wild-type *Arabidopsis thaliana* var. *columbia* by the floral dip method [59]. Transgenic *Arabidopsis* seeds were selected on solid Murashige and Skoog (MS) medium supplemented with 25 µg/L hygromycin (hyg). Independent hyg-resistant transgenic plants were further confirmed by PCR amplifications of the insertion cDNA.

### Germination and tolerance analysis of *AhGLPs* in transgenic *Arabidopsis*

For *in vivo* salt-tolerance arrays, seeds from wild type and *AhGLPs* transgenic *Arabidopsis* were surface-sterilized as described by Clough and Bent [59] and sown on MS plates plus 2% sucrose containing 0, 50 and 100 mM NaCl, respectively. After stratification at 4°C for 3 days, plates were transferred in a growth chamber (100 µE m^−2^ s^−1^, 16 hr light/8 hr dark, 22°C). Germination (scored based on radicle emergence) was monitored daily for 6 days. After 100 mM NaCl treatment for 15 days, the seedlings were photographed and the tolerance of different transgenic *Arabidopsis* lines was observed. All the experiments were performed in duplicates. The mean of technical replicates was presented in the results. T-test analysis was performed to determine the statistical significance.

### Subcellular localization of *AhGLPs::GFP* in onion epidermal cells

The coding sequences of *AhGLP1*, *2*, *3*, *4*, *5* and *7* were amplified by PCR (Additional File 2) and fused to the 3′ region of the *GFP* gene, respectively. The *AhGLP*-*GFP* fusion genes were subcloned into the pCAMBIA1301 vector, for the expression under the control of CaMV35S promoter. These *AhGLP-GFP* fusion constructs and empty vector control were introduced into onion epidermal cells through the *Agrobacterium*-mediated system. The obtained cells were cultured on 1/2 MS medium at 26°C in darkness for 24 h. Subcellular localization of AhGLP proteins in onion (*Allium cepa*) epidermal cells indicated by the GFP signal was observed under fluorescence microscopy (Axio Observer A1, Zeiss, Germany). All transient expression assays were repeated at least three times.

## Supporting Information

Figure S1 **Homology matrix of predicted amino acid sequences of AhGLP family.**
(DOC)

Table S1 **Primers used to quantify transcripts from the peanut** ***GLP*** **family and** ***18S*** **genes by qRT-PCR.**
(DOC)

Table S2 **Primers used for** ***AhGLP*** **s transgenic and subcellular localization analysis.**
(DOC)
