# Supplementary material for: Characterization of Peanut Germin-Like Proteins, AhGLPs in Plant Development and Defense
Source: PLoS One. 2013 Apr 23;8(4):e61722. doi: 10.1371/journal.pone.0061722 (PMC3633998; doi:10.1371/journal.pone.0061722)
Supplement: Figure S1 — Homology matrix of predicted amino acid sequences of AhGLP family. (DOC) [file pone.0061722.s001.doc]

| **AhGLP1** | **100** |  |  |  |  |  |  |  |  |  |  |
| --- | --- | --- | --- | --- | --- | --- | --- | --- | --- | --- | --- |
| **AhGLP2** | 35.2 | **100** |  |  |  |  |  |  |  |  |  |
| **AhGLP3a** | 35.7 | 46.7 | **100** |  |  |  |  |  |  |  |  |
| **AhGLP3b** | 35.2 | 45.8 | 99.1 | **100** |  |  |  |  |  |  |  |
| **AhGLP4** | 46.3 | 32.4 | 32.2 | 31.7 | **100** |  |  |  |  |  |  |
| **AhGLP5a** | 56.3 | 32.5 | 34.6 | 34.1 | 49.5 | **100** |  |  |  |  |  |
| **AhGLP5b** | 56.9 | 31.4 | 35.4 | 35.0 | 48.8 | 98.6 | **100** |  |  |  |  |
| **AhGLP6** | 37.6 | 61.5 | 50.0 | 49.0 | 32.7 | 34.8 | 33.7 | **100** |  |  |  |
| **AhGLP7a** | 50.5 | 32.7 | 34.0 | 33.5 | 63.3 | 51.9 | 51.2 | 32.0 | **100** |  |  |
| **AhGLP7b** | 50.2 | 33.0 | 33.3 | 32.9 | 63.5 | 51.7 | 51.0 | 31.6 | 97.7 | **100** |  |
| **AhGLP8** | 35.9 | 63.3 | 49.6 | 48.7 | 27.3 | 34.5 | 35.4 | 73.8 | 32.5 | 30.8 | **100** |
|  | **AhGLP1** | **AhGLP2** | **AhGLP3a** | **AhGLP3b** | **AhGLP4** | **AhGLP5a** | **AhGLP5b** | **AhGLP6** | **AhGLP7a** | **AhGLP7b** | **AhGLP8** |
