# Supplementary material for: Characterization of Peanut Germin-Like Proteins, AhGLPs in Plant Development and Defense
Source: PLoS One. 2013 Apr 23;8(4):e61722. doi: 10.1371/journal.pone.0061722 (PMC3633998; doi:10.1371/journal.pone.0061722)
Supplement: Table S1 — Primers used to quantify transcripts from the peanut GLP family and 18S genes by qRT-PCR. (DOC) [file pone.0061722.s002.doc]

**Table S1. Primers used to quantify transcripts from the peanut *GLP* family and *18S* genes by** qRT-PCR.

| **Genes** | **Primers for qRT-PCR** | **Length (bp)** |
| --- | --- | --- |
| *AhGLP1* | F: CGGAGACACCAACAACACCTT  R: TCTAACCTTGCCGCCGATACT | 99 |
| *AhGLP2* | F: GTTCCATTGTAACAGGAGCCA  R: TGAGTCCACCTGGGGCATA | 96 |
| *AhGLP3* | F: GCCTCTTTTGCCTCTGCCTA  R: GACGGCATCAAAGACGCTGT | 81 |
| *AhGLP4* | F: ATTTTCTTCCTCTTCGCCCTT  R: GACTTGGCCGGTTTACAGGGA | 119 |
| *AhGLP5* | F: AAAATGAAGATGGTTCTCGCC  R: CACAGAAATCTACTACGGAAGCC | 82 |
| *AhGLP6* | F: GGCTTCTGATCCTGAACCACT  R: AGGCTTCCCTATGCCACTAAA | 136 |
| *AhGLP7* | F: GGGAGCACAAACCCCTGAA  R: TGGAAGAGTTGGATGAGGGC | 116 |
| *AhGLP8* | F: CAGCAGTGCCATCTCCTTTG  R: GCACGCAAATCCATTCACC | 112 |
| *18S* | F: GTTCCACTATGTTCCCAGGCA  R: CTTCCTCTCTGGTGGTGCTACA | 104 |
| *PR3* | F: TGGCAAACGCTACTACGGAAG  R: AAGCGATCACTGCGTCGTTG | 131 |
| *PR4* | F: AATTGTTGGGGAAGTGGGCC  R: TCCCAATTATTCTGCGCCGG | 95 |
| *PR5* | F: GTCTCGCCTCCAGTCAAACG  R: TTCAAGCCCTGCTCCAGAAA | 147 |
| *CHS* | F: TGCGACAAGTCGACAATTCG  R: TAGGCAAAGAAGCGGCAGTG | 156 |
| *DFR* | F: CAAACGCCAAGACGCTACTCA  R: CACTGTCGGCTTTATCACTTCG | 158 |
| *3GT* | F: GGCCAGAGGTCGAGACCATT  R: GACGCAGCGCTAACGATGTT | 151 |
| *actin* | F: GTTTGCGACAATGGAACTGGA  R: GATTGAGCTTCATCGCCAACA | 155 |

*18S* (18S rRNA) and *actin* (*Atactin1*) are the internal control gene of peanut and *Arabidopsis* respectively. *CHS*: *Chalcone synthase*; *DFR*: *Dihydroflavonol 4-reductase*; *3GT*: *UDP-glucoeseflavonoid-3-oxy-glucosytransferase*.
