# Supplementary material for: Characterization of Peanut Germin-Like Proteins, AhGLPs in Plant Development and Defense
Source: PLoS One. 2013 Apr 23;8(4):e61722. doi: 10.1371/journal.pone.0061722 (PMC3633998; doi:10.1371/journal.pone.0061722)
Supplement: Table S2 — Primers used for AhGLP s transgenic and subcellular localization analysis. (DOC) [file pone.0061722.s003.doc]

**Table S2 Primers used for *AhGLP*s transgenic and subcellular localization** analysis.

| **Primer No.** | **Sense primer sequences (5’-3’)** | **Antisense primer sequences (5’-3’) for transgenic analysis** | **Antisense primer sequences (5’-3’) for subcellular localization** |
| --- | --- | --- | --- |
| ***AhGLP1*** | **ggatccATG**AAGAGCATGAGGATT | **aagcttCTA**CATAGACATATGGTTATTCA | **ctgcag**CATAGACATATGGTTATTCA |
| ***AhGLP2*** | **ggatccATG**AAGTTCATAGGCTCAG | **aagcttTTA**CTTCTTGGGAGCAAGCC | **ctgcag**CTTCTTGGGAGCAAGCCTAG |
| ***AhGLP3*** | **ggatccATG**AAAGCTACATACTTGCT | **ctgcagCTAGTTGTTATCATACCAA** | **ctgcag**GTTGTTATCATACCAAAACT |
| ***AhGLP4*** | **ggatccATG**CAAAATGTTTACCAT | **aagcttCTATCCTGGACCACCAAAA** | **ctgcag**TCCTGGACCACCAAAAACAC |
| ***AhGLP5*** | **ggatcc**AA**ATG**AAGATGGTTCTCG | **aagcTTAACTTGAGCCTCCAAGCAC** | **tctaga**ACTTGAGCCTCCAAGCACA |
| ***AhGLP7*** | **ggatccATG**TCATTGATTGATTCATT | **ctgcagAATTATTATCCACTGCCAC** | **ctgcag**TCCACTGCCACCAAAAATA |

All the sense primers were inserted with BamHI recognition site at the 5’. The antisense primers (AhGLP1, 2, 4 and 5) used for transgenic analysis, were inserted with HindIII recognition site at 5’, but AhGLP3 and 7 with Pst I recognition site at 5’. Fro subcellular localization analysis, only antisense primer of AhGLP5 was inserted with XbaI recognition site, other were designed PstI recognition sites.

| **AhGLP1** | **100** |  |  |  |  |  |  |  |  |  |  |
| --- | --- | --- | --- | --- | --- | --- | --- | --- | --- | --- | --- |
| **AhGLP2** | 35.2 | **100** |  |  |  |  |  |  |  |  |  |
| **AhGLP3a** | 35.7 | 46.7 | **100** |  |  |  |  |  |  |  |  |
| **AhGLP3b** | 35.2 | 45.8 | 99.1 | **100** |  |  |  |  |  |  |  |
| **AhGLP4** | 46.3 | 32.4 | 32.2 | 31.7 | **100** |  |  |  |  |  |  |
| **AhGLP5a** | 56.3 | 32.5 | 34.6 | 34.1 | 49.5 | **100** |  |  |  |  |  |
| **AhGLP5b** | 56.9 | 31.4 | 35.4 | 35.0 | 48.8 | 98.6 | **100** |  |  |  |  |
| **AhGLP6** | 37.6 | 61.5 | 50.0 | 49.0 | 32.7 | 34.8 | 33.7 | **100** |  |  |  |
| **AhGLP7a** | 50.5 | 32.7 | 34.0 | 33.5 | 63.3 | 51.9 | 51.2 | 32.0 | **100** |  |  |
| **AhGLP7b** | 50.2 | 33.0 | 33.3 | 32.9 | 63.5 | 51.7 | 51.0 | 31.6 | 97.7 | **100** |  |
| **AhGLP8** | 35.9 | 63.3 | 49.6 | 48.7 | 27.3 | 34.5 | 35.4 | 73.8 | 32.5 | 30.8 | **100** |
|  | **AhGLP1** | **AhGLP2** | **AhGLP3a** | **AhGLP3b** | **AhGLP4** | **AhGLP5a** | **AhGLP5b** | **AhGLP6** | **AhGLP7a** | **AhGLP7b** | **AhGLP8** |

**Supplemental Fig 3. Homology matrix of 11 sequences.**
